# Innovations in microsurgery: The role of non‐invasive blood perfusion imaging—A review and framework

**DOI:** 10.1002/cap.70042

**Published:** 2026-01-26

**Authors:** Amanda B. Rodriguez, Oliver D. Kripfgans, Diego Velásquez‐Plata, Hsun‐Liang Chan

**Affiliations:** ^1^ Department of Periodontics College of Dentistry University of Illinois Chicago Chicago Illinois USA; ^2^ Department of Radiology Michigan Medicine University of Michigan Ann Arbor Michigan USA; ^3^ Seven Lakes Periodontics Private Practice Fenton Michigan USA; ^4^ Division of Periodontology College of Dentistry The Ohio State University Columbus Ohio USA

**Keywords:** laser speckle, microcirculation, non‐invasive, ultrasound, vascularization, wound healing

## Abstract

**Background:**

This review evaluates current literature on non‐invasive blood perfusion imaging in periodontology, with a focus on applications in periodontal microsurgery.

**Methods:**

A hypothesis‐building review was conducted by two reviewers from January 2022 to April 2025 across multiple databases.

**Results:**

Laser speckle contrast imaging (LSCI) and ultrasound (US) emerged as the most promising modalities. LSCI measures superficial gingival perfusion with high spatial resolution, demonstrating sensitivity to vascular dynamics and sex‐related differences during tissue compression. US, using B‐mode and color flow‐based imaging, provides both anatomical and functional assessment of gingival and peri‐implant tissues, showing correlations with inflammation and healing outcomes. Both techniques have been validated in clinical and preclinical settings as reliable tools for assessing perfusion during regenerative procedures. A clinical case illustrates the use of LSCI and US to evaluate blood perfusion in a grafted alveolar ridge.

**Conclusions:**

Blood perfusion is a key factor in wound healing, heavily influenced by surgical trauma and flap tension. Microsurgical techniques may improve outcomes by minimizing soft tissue trauma and preserving vascular integrity during flap procedures. LSCI and US together could offer complementary, real‐time, chairside imaging to monitor perfusion, advancing personalized treatment strategies and improving prediction of healing outcomes in periodontal and peri‐implant therapy.

**Key points:**

- Laser speckle contrast imaging (LSCI) and ultrasound (US) emerge as the leading non‐invasive modalities for assessing periodontal tissue perfusion, with LSCI capturing superficial microvascular flow and US providing complementary anatomical and functional detail.
- Evidence from recent clinical and preclinical studies, and an illustrative case example, demonstrates that both technologies reliably evaluate perfusion during regenerative and microsurgical procedures, including grafted alveolar ridges.
- Because adequate perfusion is essential for wound healing, integrating LSCI and US into periodontal practice may support real‐time, personalized, data‐driven clinical decision making and help optimize outcomes in periodontal and peri‐implant therapy.

**Plain language summary:**

This review examines the use of non‐invasive imaging technologies to assess blood flow (perfusion) in periodontal tissues, with a focus on their application in periodontal microsurgery. A literature search covering studies published from January 2022 to April 2025 identified laser speckle contrast imaging (LSCI) and ultrasound (US) as the most promising modalities. LSCI provides high‐resolution assessment of superficial gingival perfusion and is sensitive to vascular changes, while US, using B‐mode and color Doppler, offers both anatomical and functional insights into gingival and peri‐implant tissues. Both techniques have demonstrated clinical and preclinical reliability in evaluating perfusion during regenerative procedures, as seen in the literature review. A case example presented by the authors illustrates how LSCI and US can be used in combination to assess blood flow in a grafted alveolar ridge. Adequate perfusion is essential for wound healing and may be compromised by surgical trauma and flap tension. Microsurgical techniques, which aim to minimize tissue trauma and preserve vascular integrity, may enhance healing outcomes. The integration of LSCI and US into clinical practice could enable real‐time, chairside assessment of tissue perfusion, supporting more personalized, data‐driven decision making in periodontal and peri‐implant therapies.

## INTRODUCTION

Microsurgery, especially with the assistance of the operating microscope (OM), has gained critical attention in the field of periodontics and implant dentistry.^1^, ^2^, ^3^, ^4^, ^5^ Three areas that benefit the most are the periodontal and mucogingival plastic reconstruction, and maxillary sinus augmentation. Primary wound closure, essential for predictable periodontal regeneration, has been achieved more consistently with microsurgical techniques, including refined flap designs, biologics, and the use of the OM.^3^, ^5^, ^6^ After microsurgical regeneration, teeth with an initially hopeless prognosis gained significant clinical attachment and maintained periodontal stability over 10 years.^6^ Positive outcomes have also been reported by using the OM/surgical loupes for root coverage procedures. A systematic review summarized that microsurgery had 30% greater chance of achieving complete root coverage than macrosurgery.^3^ The OM has the potential to reduce postoperative morbidity and improve the clinical outcome of sinus augmentation by applying the minimally invasive approach and allowing early diagnosis and management of perforation of the Schneiderian membrane.^7^

Periodontal microsurgery represents an evolution of foundational surgical techniques, refined through enhanced visual precision made possible by the OM.^5^, ^8^ While traditionally valued for its technical benefits, including superior magnification, consistent illumination, and compatibility with fine instruments and sutures, the OM's role is often framed primarily within this functional context.^8^ These advantages must interact with the biologic system, that is, the periodontium in our case for the clinical outcome to be evident. The most feasible assumption is that OM‐assisted procedures modulate the wound healing process.^9^, ^10^, ^11^, ^12^, ^13^ It is likely that OM‐assisted surgeries can sustain sufficient blood perfusion for optimal healing by maximizing wound stability and preserving soft tissue integrity, both leading to subsequent healing steps that primarily rely on blood perfusion.^4^ Therefore, it becomes clear that evaluating blood perfusion would be a crucial step to unveil the mechanisms with which OM‐assisted procedures enhance clinical outcomes.

Blood perfusion imaging modalities have evolved over the past decades, primarily based on optical,^12^, ^14^, ^15^, ^16^, ^17^ or ultrasound (US) means.^18^ For optical methods, it starts with intravital video microscopy for preclinical studies.^19^, ^20^ It requires the injection of fluorescence dye, the invasive preparation of an imaging window, and maximal positional stability. The primary mechanism is based on the illumination of moving red blood cells (RBCs) that are imaged through intravital microscopy to image periosteal microcirculation (penetration depth: approximately 250 µm; Zeiss Axiotech Vario 100HD microscope; 100‐W HBO mercury lamp; Acroplan 20×/0.5 N.A. W, Carl Zeiss GmbH).^19^ Video capillaroscopy was then developed for easily accessible anatomical locations, such as the nailbed and the sublingual site.^21^, ^22^ Further advances, such as orthogonal spectral polarization^23^ and sidestream dark field imaging,^24^ aim to increase image contrast, miniaturize device size for more clinical applications, and reduce costs. These four methods can provide morphologic (capillary size/diameter) as well as functional (blood flow velocity in mm/s) evaluation of capillaries. US, being able to estimate blood perfusion by calculating the phase shift changes of the sound waves after interacting with RBC, has been used extensively in medicine (color flow and pulse‐wave mode).^25^, ^26^ Recently, laser speckle contrast imaging (LSCI) has been introduced by computing speckle pattern changes of the laser scattered by RBC to estimate blood flow.^17^, ^27^, ^28^, ^29^, ^30^ For an overview of perfusion imaging methods, see ref.^31^ Despite the promise of LSCI and US in evaluating tissue perfusion, there is currently no direct empirical evidence supporting their application specifically in periodontal microsurgery. Although US has demonstrated utility in imaging soft tissue perfusion and inflammation, much of the supporting evidence is derived from animal models, case studies, or early‐stage clinical research.^32^, ^33^, ^34^ Therefore, the primary aim is to review the current periodontal literature on the use of blood perfusion imaging modalities and if any modalities have also been used to evaluate wound healing of periodontal microsurgeries.

## MATERIALS AND METHODS

### Literature review

A hypothesis‐building literature review was conducted by two reviewers instead of a systematic review. Although hypothesis‐building reviews carry an inherent risk of bias, we chose this approach as it allows for timely assessment, supports reproducibility by readers, and builds upon early‐stage research and conceptual frameworks to guide future empirical testing.^35^ An electronic search was conducted by two reviewers (A.R. and O.K.) from January 2022 to April 2025 in the following databases: NLM PubMed, Embase, EBSCOhost CINAHL, EBSCOhost Dentistry and Oral Sciences Source, and Wiley Cochrane Central Register of Controlled Trials. The working PubMed search was as follows: ((((periodontal surgery) AND (periodontal)) AND (gingiva)) OR (oral mucosa) OR (soft tissue) OR (alveolar bone) AND (non invasive) AND (tissue perfusion) OR (gingival blood flow) OR (blood flow) OR (perfusion) OR (diagnostic) AND (dental)) AND (periodontium). A second electronic search was performed to include the following terms: (((non invasive) AND (imaging)) AND (tissue perfusion)) AND (periodontal microsurgery) AND (microsurgery). The exploratory hypothesis proposes that emerging non‐invasive blood perfusion imaging modalities, such as LSCI and US, may provide complementary insights into vascular dynamics during periodontal and peri‐implant procedures. These technologies have the potential to enhance clinical understanding of tissue perfusion, thereby supporting their integration into clinical practice to optimize healing and improve surgical outcomes. Inclusion criteria encompassed studies involving human subjects or pre‐clinical in vivo models that utilized imaging technologies to quantify blood circulation in oral or periodontal tissues. Exclusion criteria included ex vivo studies, review articles (including workshop consensus reports), retrospective studies, and publications not available in English.

### Clinical case

The included clinical case was collected from a feasibility study approved by the University of Michigan Medical School IRB (HUM00203875), following the Helsinki Declaration, with written informed consent. The study was funded by the NIH (1‐R56‐DE‐030872‐01). This case is an exploratory example to illustrate potential applications and encourage further research, rather than to imply established clinical effectiveness.

## RESULTS

This review identified LSCI and US as the most applied non‐invasive perfusion imaging techniques. Both will be discussed for their imaging principles, indications, and clinical implications.

### Laser speckle contrast imaging

#### Principle

**FIGURE 1:**
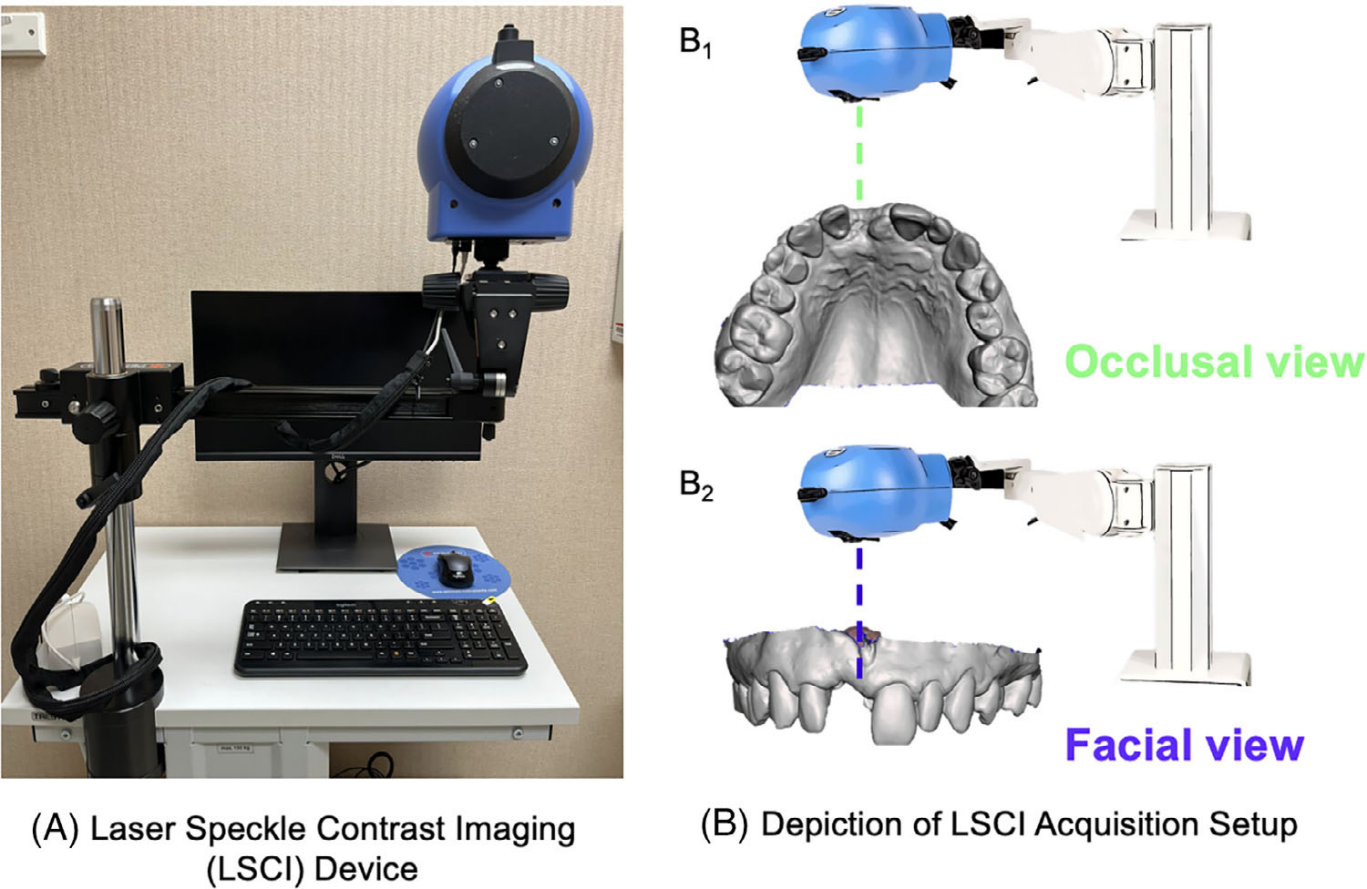
Laser speckle contrast imaging (LSCI) was performed using the PeriCam PSI HR system (Perimed), which provides up to 10 µm spatial resolution and a maximum temporal resolution of 113 frames per second (fps) across a 1.8 × 2.9 cm field of view. Image recordings were acquired at 25 fps for a duration of 30 s (A). The system is composed of a laser emitter/receiver, a computer processor, and a monitor. The positioning of the LSCI laser probe for image acquisition is illustrated for both occlusal (B_1_) and facial (B_2_) views (B).

LSCI measures mucosal or skin blood flow.^28^ LSCI has high spatial (∼10 µm) and temporal (113 frames per second [fps]) resolution, outputting arbitrary color‐coded perfusion intensity as two‐dimensional (2D) anatomical images.^29^, ^30^ LSCI quantifies superficial tissue perfusion (∼1 mm) by analyzing the speckle pattern of scattered light using an invisible (infrared) laser (wavelengths ∼785 nm) that is scanned over a 2D region of interest (Figure 1).^36^ The light reflected from stationary objects creates a stationary speckle pattern, whereas moving cells cause a dynamic speckle pattern that blurs in proportion to their speed.^27^, ^36^ This technique provides the velocity of the moving targets, in this case RBC, across a 2D surface and therefore the relative vascular density and blood volume could be estimated. Blood flow velocity distributions are presented on a monitor using false‐color encoding in perfusion units (PU): blue‒cyan for low perfusion, green‒yellow for moderate perfusion, and orange‒red for high perfusion.

The general limitations of this technique include its reliance on direct line‐of‐sight measurements, which can restrict access to certain areas of the oral cavity, such as the molars. Additionally, LSCI has a shallow penetration depth of approximately 1 mm, is highly sensitive to the working distance setting, and can be influenced by patient movement and gingival temperature. The measurement angle, which can be difficult to determine due to the curved anatomy of the maxilla and mandible, may also affect the accuracy of the blood flow values obtained^27^, ^28^, ^29^, ^30^, ^37^ (Table 1).

**TABLE 1:** Advantages and disadvantages of laser speckle contrast imaging (LSCI) and ultrasound (US).

Perfusion technique	LSCI	US
Advantages	Non‐contact Wide field of view (can cover up several adjacent teeth) Perfusion data superimposed on anatomical images	Cross‐sectional (adequate penetration depth for oral tissues with high resolution) Provide anatomical images, vessel distribution, perfusion direction, and velocity/volume of blood flow
Disadvantages	Arbitrary tissue perfusion units Limited penetration depth (∼100 µm) Large sensor (molars are hard to image, if possible)	Surrogate tissue perfusion Need contact via coupling agent Reproducible location could be difficult cross different time points. Unless a guide is used^38^

#### Periodontal literature

Several studies have evaluated gingival blood flow using LSCI.^27^, ^28^, ^29^, ^30^, ^37^, ^39^ Palombo et al.^17^ conducted a randomized controlled trial (RCT) comparing conventional (sutures and sponges) versus sutureless hemostasis for palatal epithelialized gingival graft (EGG) harvesting. Healing was assessed at 7, 14, and 30 days using LSCI. The sutureless group showed faster hyperemia resolution, with peak blood flow at day 7, although differences were not statistically significant. Fazekas et al.^27^ aimed to investigate blood flow dynamics in the gingiva at rest and under mechanical tissue compression using the LSCI method in 21 healthy patients who underwent horizontal, vertical, and papilla base compressions on the attached gingiva. LSCI was used to observe regional blood flow changes during a 5‐s occlusion and subsequent reperfusion over 20 min. Findings showed that resting blood flow was higher in the gingiva apical to the papillae compared to the mid‐buccal area. Horizontal compression induced more significant ischemia coronally than apically, with post‐occlusive hyperemia extending beyond the ischemic area. This hyperemic response was more pronounced and lasted longer in males than females.^27^ A functional study by Mikecs et al.^39^ used LSCI to measure changes in gingival blood flow in 31 healthy subjects during 30‐s compressions. Findings showed that the superior labial artery formed collateral plexuses supplying the attached gingiva, and compression of these branches caused ischemia with varying individual responses. Males exhibited higher baseline flow and larger ischemic areas compared to females. A significant correlation between ischemia and flow decrease was found. Additionally, blood flow restoration during compression was more pronounced in males than females. The study highlights how the vasculature in the upper esthetic zone could be affected by vertical incisions. Individual variations in ischemic responses could impact surgical outcomes, and men may have different vascular reactivity, which could influence healing. This technique has been validated by previous studies as it demonstrates good reproducibility in human gingiva.^28^, ^39^

An in vitro preclinical model was used to distinguish normal from osteoporotic maxilla and mandible bones.^37^ Speckle contrast and patches ratios were analyzed; the former differentiated healthy from osteoporotic tissue, while the latter showed a negative correlation with surface roughness. LSCI demonstrated potential for detecting osteoporosis in alveolar bone. Initially, Molnár et al.^29^ assessed the reliability of gingival blood flow using LSCI in seven healthy subjects. Variables such as angle of incidence, lip retraction, and mouth closure were tested and repeated after 1 week. Results confirmed that LSCI remains consistent over time, even with indirect viewing or lip retraction.^29^ In a 2019 study, Molnár et al. also evaluated healing at palatal donor sites in seven patients after connective tissue harvesting.^28^ They found elevated postoperative blood flow in secondary healing areas, with delayed normalization compared to adjacent tissue. This delay is attributed to secondary versus primary wound healing. Additionally, reperfusion time strongly correlated with healing scores.

### Ultrasound

#### Principle

**FIGURE 2:**
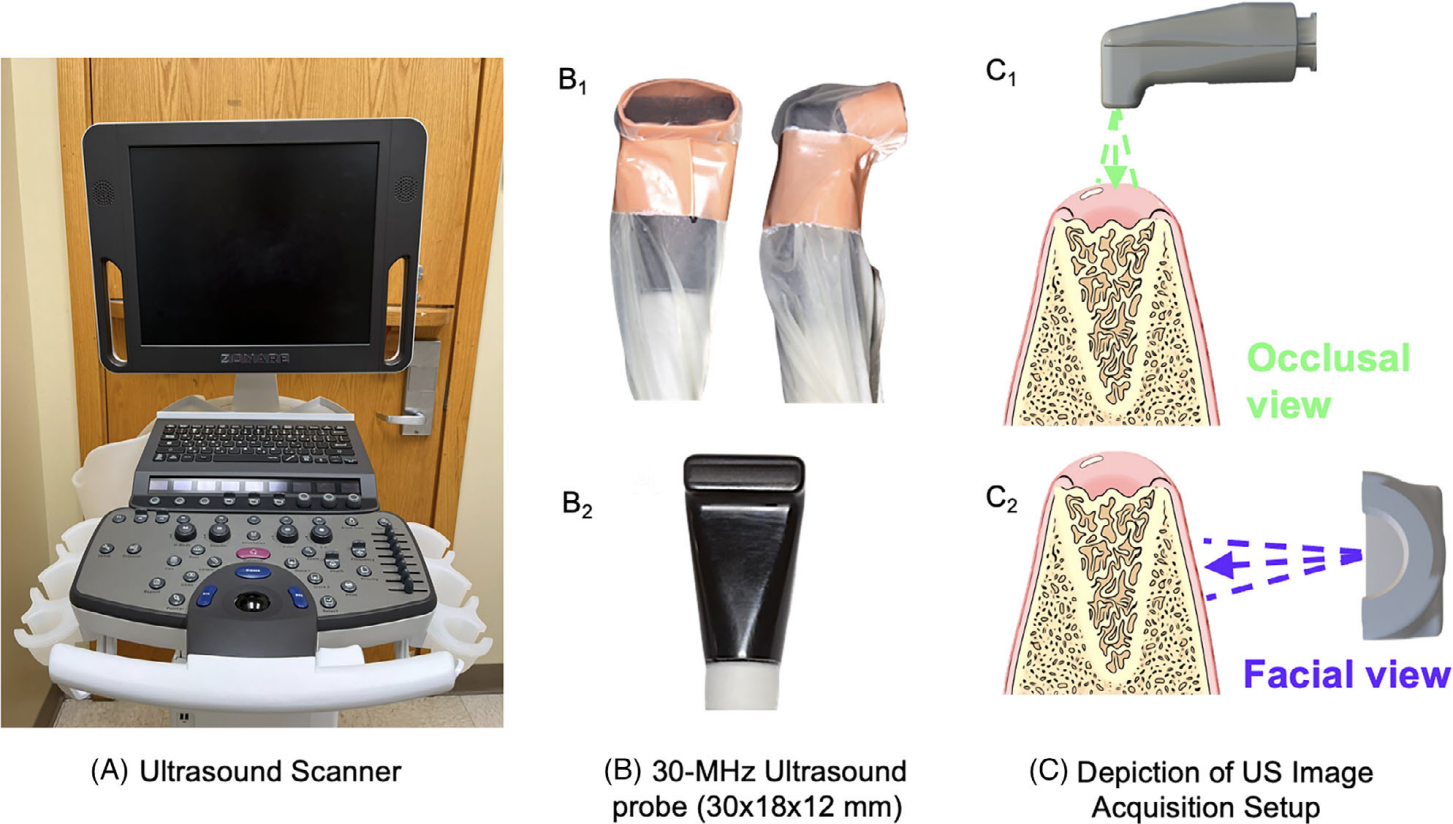
Ultrasound (US) imaging was performed using a Mindray/Zonare system (Mindray/Zonare of North America) (A), equipped with an L30‐8 linear array transducer (B). This transducer provides an axial image resolution of 64 µm and features an elevational focus of 8 mm. Its compact footprint, measuring approximately 30 mm in length, 18 mm in width, and 12 mm in thickness allows for intraoral use. Photographs (B_1_ and B_2_) of the transducer: front and side views shown with (B_1_) and without (B_2_) the protective sheath used for infection control in clinical procedures. US probe positioning was standardized for image acquisition, with specific orientations used for occlusal (C_1_) and facial (C_2_) imaging (C).

US is a cross‐sectional, non‐ionizing, and chair‐side imaging technique.^18^ It evaluates soft tissues and hard tissue surfaces based on the reflection and scattering of sound by the interaction of emitted acoustic waves with tissues.^18^, ^38^, ^40^, ^41^ US images can be obtained as anatomical images, that is, brightness mode (B‐mode), and more appealing for tissue perfusion evaluation with parameters, such as color flow velocity (CV) or color flow power (CP). B‐mode images are composed of grayscale pixels, and the intensity indicates the amount of sound scattered or reflected back depending on the anatomical site, as hypoechoic (soft tissue) or hyperechoic (hard tissues).^25^, ^33^ For perfusion analysis, the B‐mode display is overlaid with color flow pixels in which blood flow is displayed that exceed a preset (noise/wall) filter threshold. Moving RBCs scatter the incident acoustic wave and produce signals that change the radio frequency phase if the blood flow direction is non‐perpendicular to the US beam (Figure 2).^18^ Velocity images display red and blue colors, which correspond to the orientation of the detected velocity (speed) with respect to the US beam direction.^25^ Power is displayed as a single hue of red that is quantitatively proportional to the amount of moving blood within the US resolution cell associated with a given pixel.^25^, ^40^ Color flow quantification can be performed by computation of the color pixel density (CPD), depending on the mode, which can either be velocity (CPDV) or power (CPDP) weighted pixel density. Limitations of this imaging technology are the reliance on a (gel) coupling medium, potential user dependency, sensitivity to probe pressure over tissues, scanning angulation, and anatomical accessibility^18^ (Table 1).

#### Periodontal literature

US has been used over the past three decades to evaluate the oral and maxillofacial complex,^42^ periapical lesions,^43^, ^44^ periodontal and peri‐implant tissues,^25^, ^26^, ^33^, ^44^, ^45^ oral pathologies.^46^ It has proven useful for imaging soft tissue thickness and hard tissue margins,^18^, ^25^, ^45^, ^47^ and monitoring peri‐implant soft tissue healing after augmentation.^26^ Most studies used transducers with frequencies between 5 and 40 MHz,^18^ but recent progress includes a newly developed high‐frequency small form factor linear array transducer for more detailed intraoral imaging.^48^ Current case definitions for periodontal and peri‐implant diseases rely on late‐stage markers such as probing depth (PD), bleeding on probing (BOP), and bone loss,^42^ which lack sensitivity and specificity.^18^ US may enable earlier, subclinical detection and support precision therapies.^40^, ^49^, ^50^ A case demonstration introduced B‐mode and color flow imaging for peri‐implant disease diagnosis in humans,^25^ showing that US provides both anatomical and functional insights for evaluating tissue inflammation and destruction.

Tikku et al.^44^ demonstrated that B‐mode and color flow imaging (also known as color Doppler) outperformed radiography in detecting healing of periapical lesions in 15 patients after surgery, supporting their use alongside conventional imaging. A preclinical mini‐pig study has further explored US in inflammation, showing increased vascular perfusion under compression during gingivitis, particularly in males.^51^ Another study^45^ reported increased soft tissue thickness and perfusion after bacterial challenge, influenced by tooth type, sex, and possible growth effects. Woo et al.^51^ also evaluated gingival vascular response to tissue compression in eight mini‐pigs over 10 weeks. The compression increased significantly in males at weeks 2, 4, 6, and 8, and in females only at week 4, indicating stronger inflammatory responses in males. This supports US as a tool for assessing gingival inflammation.

A pilot human study^52^ used US to monitor healing at implant and palatal donor sites after coronally advanced flap (CAF) and connective tissue graft (CTG) in five patients. CV and CP increased at 1 week and 1 month, peaking at 199.25% above baseline, then declined by 6 and 12 months. CP rose early at donor and foramen sites. Another study^53^ found a correlation between 7‐day postoperative biomarkers and US perfusion measures. In an RCT,^26^ tunneling (TUN) yielded better interproximal perfusion and early graft vascularization than CAF, correlating with improved soft tissue outcomes at 12 months. US shows value across planning, intraoperative, and postoperative stages,^18^ complementing radiographs, cone beam computed tomography (CBCT), and clinical exams. Its strengths—soft tissue contrast, bone surface details, and non‐ionizing use—are driving broader adoption in dentistry.^18^, ^42^, ^44^, ^48^, ^54^

### An example of applying US and LSCI in clinical research

**FIGURE 3:**
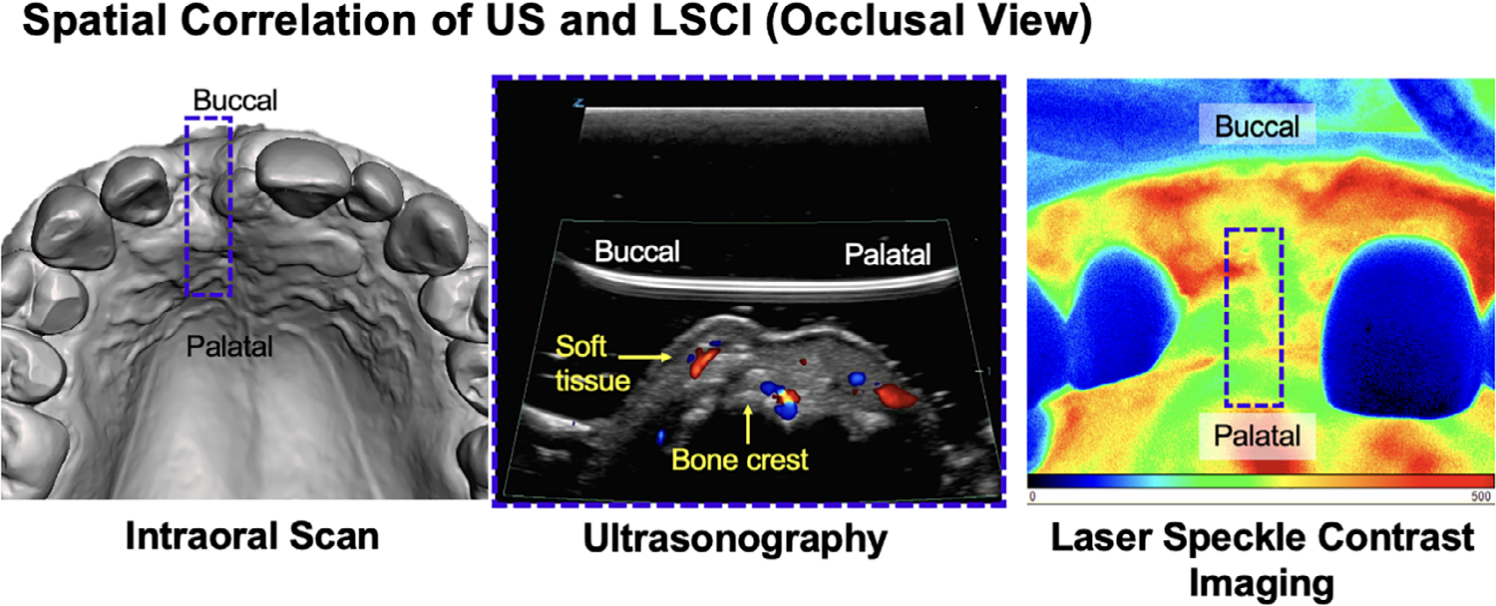
Spatial correlation of the edentulous ridge in anterior maxillary site (#8) using ultrasound (US) and laser speckle contrast imaging (LSCI) in an occlusal view. This figure demonstrates the complimentary benefits of combing the two techniques for evaluation of blood perfusion because US and LSCI offer cross‐sectional/deep‐perfusion and projection/superficial‒perfusion views, respectively.

**FIGURE 4:**
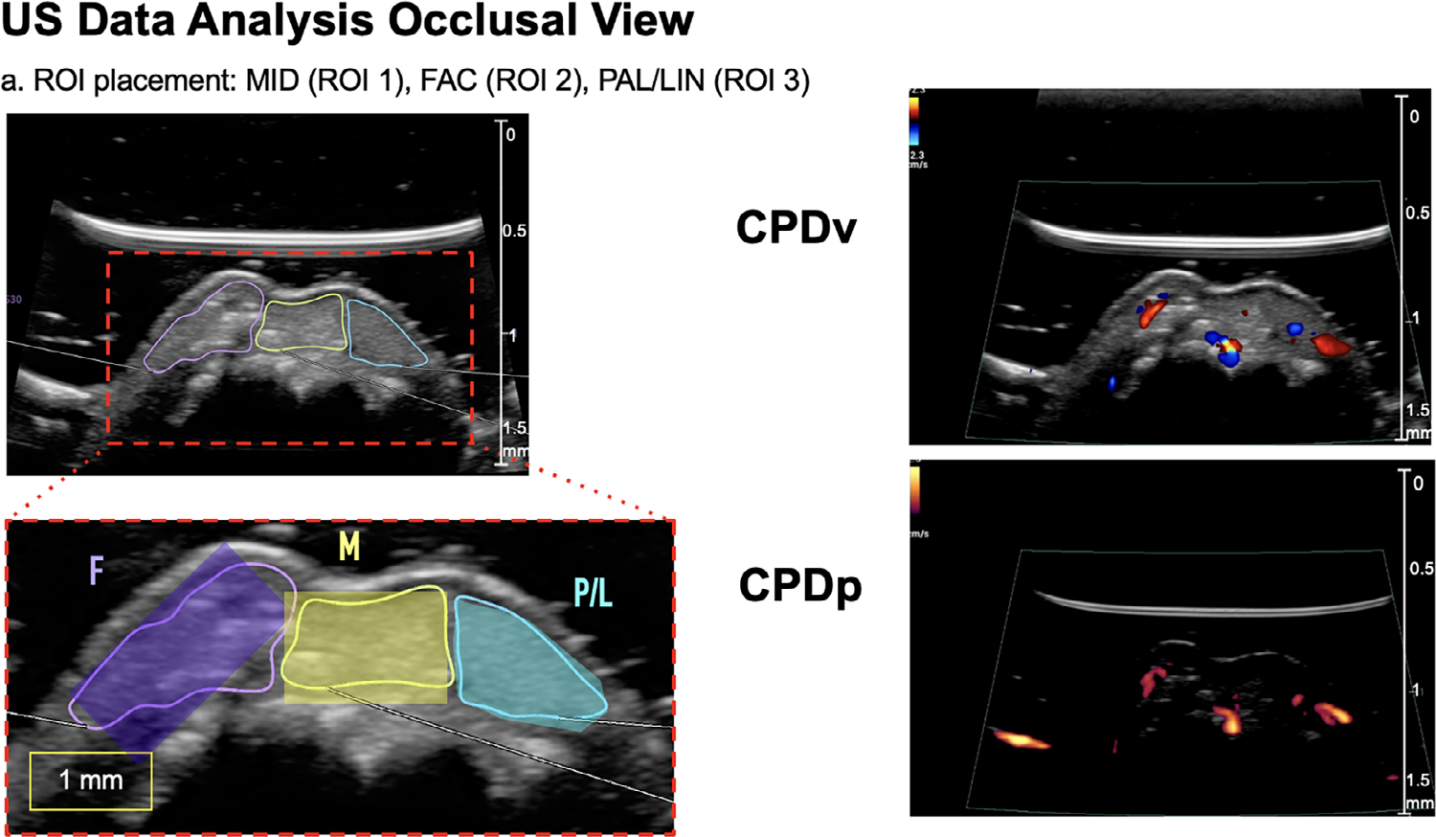
Edentulous ridge at 5 months after alveolar ridge augmentation was imaged with ultrasound (US) using a linear array (L30‐8, Mindray/Zonare) with 64‐µm axial resolution, second harmonic imaging (12‒24 MHz), and spatial compounding. B‐mode images (left) were captured. Color flow velocity (right top) and power images (right bottom) were obtained from occlusal view, with color flow recorded as a 90 dB B‐mode gain cine‐loop and power mode as a 40 dB B‐mode gain cine‐loop. Three regions of interest (ROIs) were defined as an example: mid‐occlusal (ROI 1), facial (ROI 2), and palatal/lingual (ROI 3), and color pixel density was calculated to indicate blood perfusion.

**FIGURE 5:**
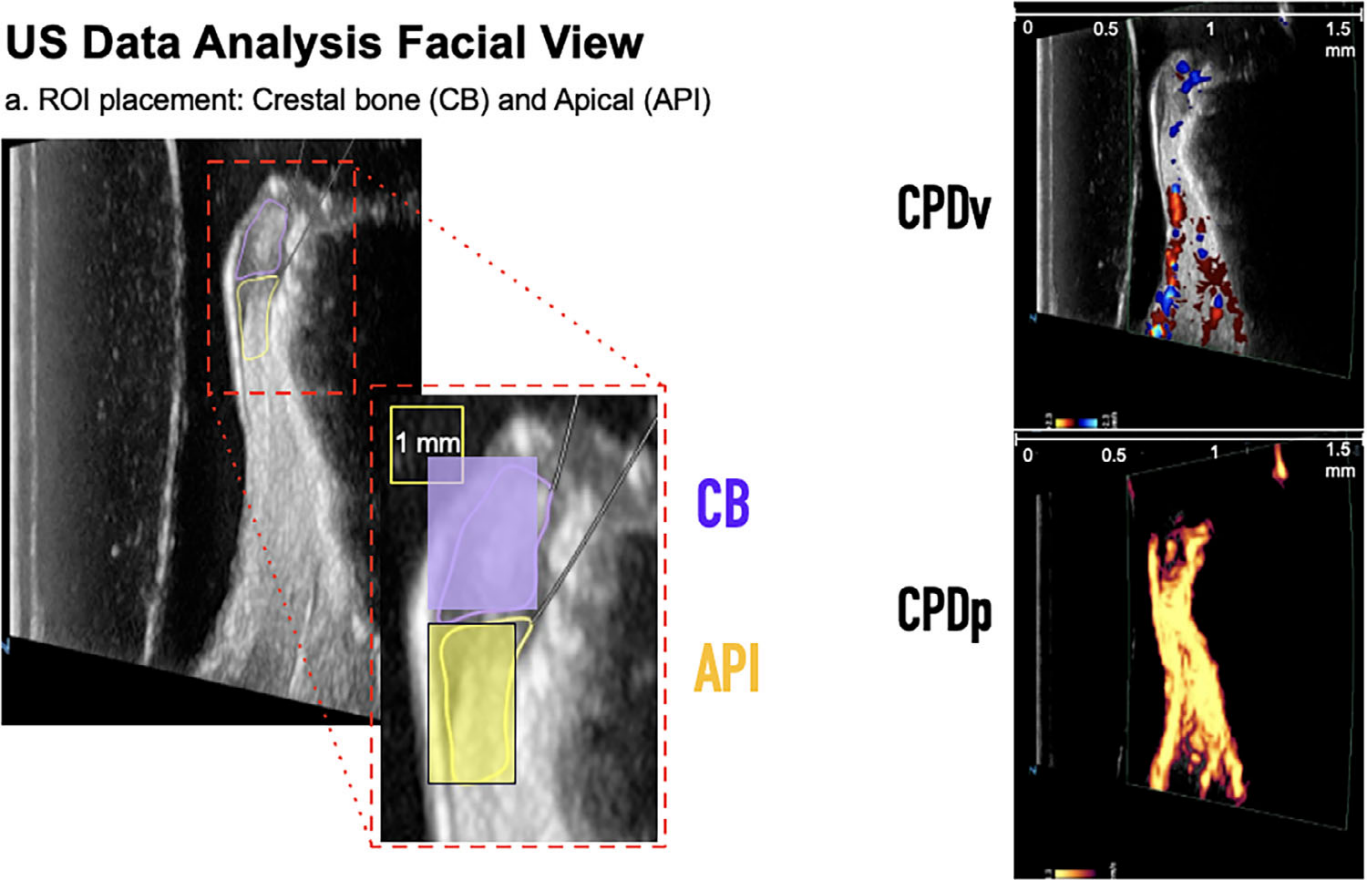
Same case was imaged from the facial view with ultrasound (US). B‐mode images (left), color flow velocity (right top) and power images (right bottom) were obtained from facial views, with color flow velocity recorded at 90 dB B‐mode gain cine‐loop and color flow power mode at 40 dB B‐mode gain cine‐loop. Two regions of interest (ROIs) were defined as an example: crestal bone (CB) and apical (API), and color pixel density was calculated to indicate blood perfusion.

**FIGURE 6:**
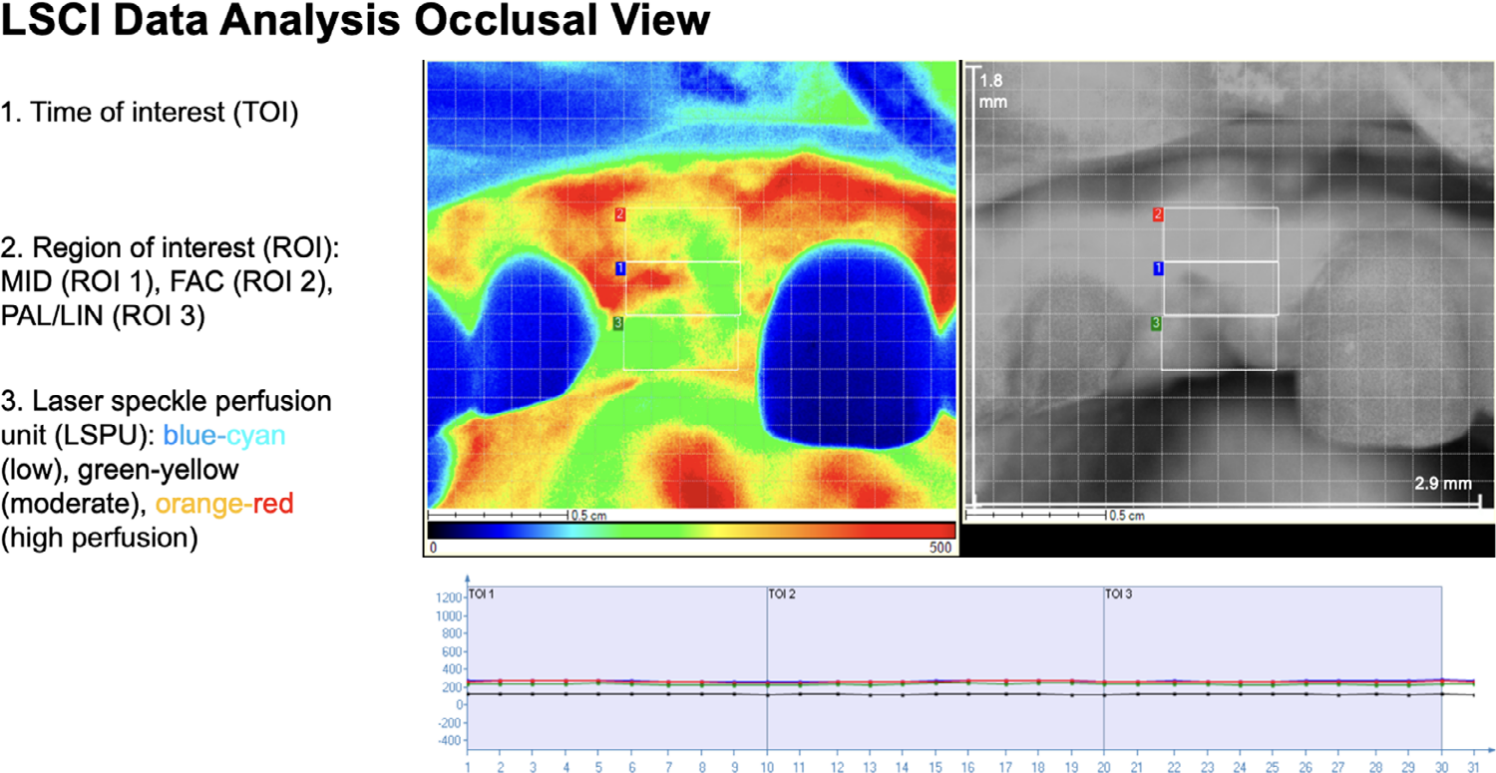
Laser speckle contrast imaging (LSCI) data acquisition on the occlusal view. LSCI assessed blood perfusion in real‐time with a spatial resolution of 10 µm and a temporal resolution of 113 frames per second (fps), using a 1.8 × 2.9 cm field of view. In this case, recordings lasted 30 s were divided into three times of interest (TOI) at 25 fps (*x*‐axis). Measurements were expressed in arbitrary perfusion units (PU) and displayed in color‐coded images (*y*‐axis): blue‒cyan for low perfusion, green‒yellow for moderate, and orange‒red for high perfusion. Three regions of interest (ROIs) were defined: mid‐occlusal (MID), facial (FAC), and palatal/lingual (PAL/LIN), and the blood perfusion in the chart was shown as ∼220 PU (blue, green, and red lines), respectively. The black line shows the average perfusion of the whole field of view.

**FIGURE 7:**
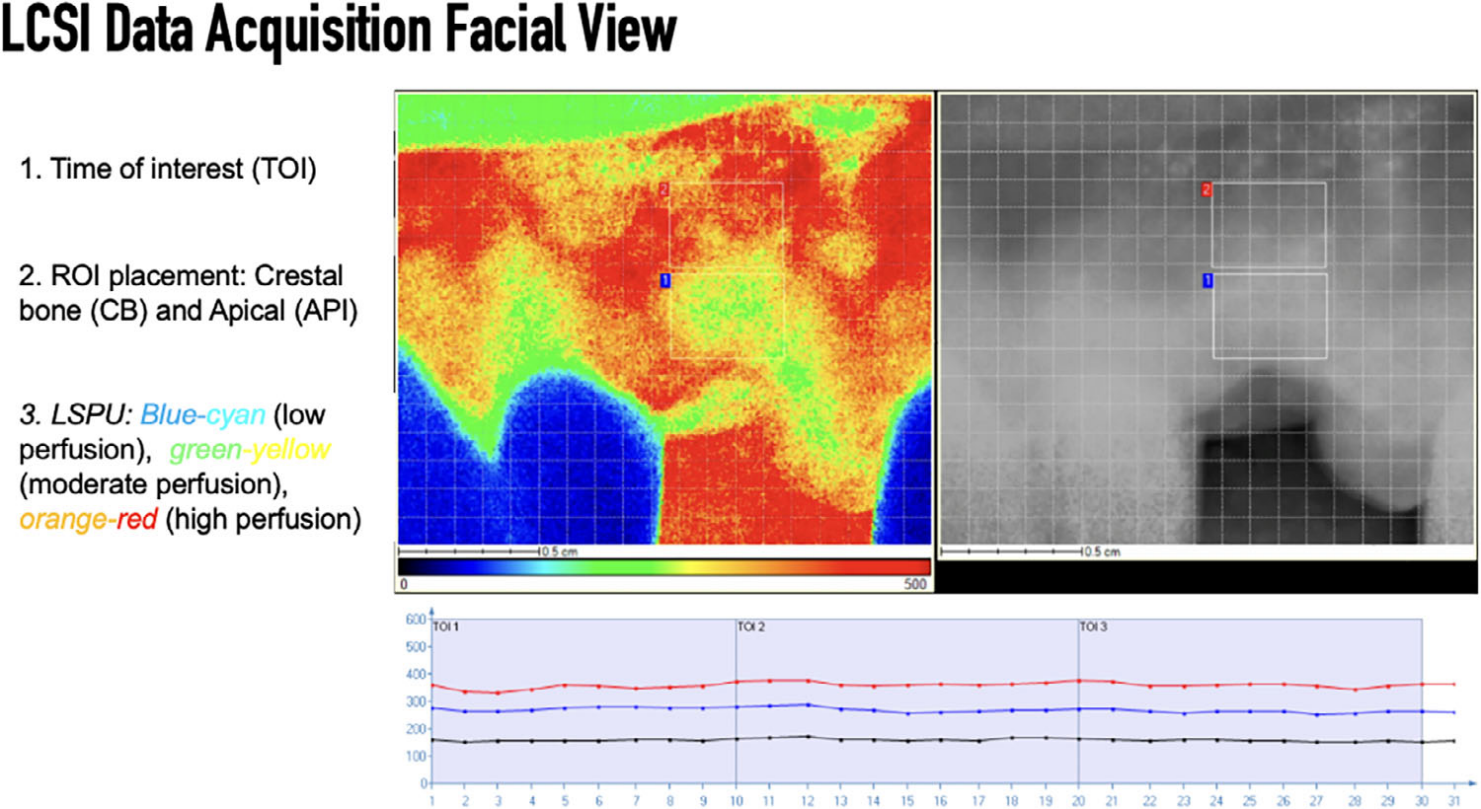
Example of facial view on laser speckle contrast imaging (LSCI) data acquisition with the same settings in Figure 6. Two regions of interest (ROIs) were defined: mid‐facial crestal bone (CB) and apical (API) regions, and the average blood perfusion in the chart was ∼280 PU (red line) and 360 PU (blue line) (*y*‐axis), respectively, over a 30‐s period (*x*‐axis). The black line shows the average perfusion of the whole field of view.

A clinical case (Figure 3) illustrates the combined use of US and LSCI in a 30‐year‐old female post‐extraction with alveolar ridge preservation, scheduled for implant placement. US imaging, as previously described,^38^, ^47^ was performed using a high‐resolution linear transducer (L30‐8, Mindray/Zonare, 64‐µm axial resolution, 12‒24 MHz second harmonic, three‐angle spatial compounding) (Figures 4 and 5). B‐mode images were guided by a 3D‐registered stent based on intraoral and CBCT scans. CV and CP images were captured from the occlusal view. Simultaneously, perfusion was assessed with LSCI (PeriCam PSI HR, Perimed) offering up to 10 µm spatial and 113 fps temporal resolution over a 1.8 × 2.9 cm field (Figures 6 and 7). Recordings were 30 s at 25 fps, producing color‐coded PU: blue‒cyan (low), green‒yellow (moderate), and orange‒red (high). Regions of interest (ROIs) included mid‐occlusal (ROI 1), facial (ROI 2), palatal/lingual (ROI 3), mid‐facial crestal bone (CB), and apical (API) regions. For spatial correlation, ROIs were standardized across both modalities (Figure 3). LSCI data were analyzed using relative PU. Both techniques revealed lower perfusion in the mid‐occlusal crest versus facial and palatal/lingual ROIs, reflecting the apico‐coronal vascular pattern. Similarly, lower CB perfusion was observed compared to the API region, consistent with higher mucosal vascularity found in the literature. This clinical case illustrates a potential diagnostic framework that integrates and correlates US and LSCI in the context of periodontal microsurgery, offering a foundation for future research.

### LSCI and US for microsurgical evaluation

Literature searches reveal no hits pertinent to using LSCI or US on microsurgical periodontal wound healing evaluation. A research hypothesis and rationale will be constructed in the “Discussion” section for future studies.

## DISCUSSION

### Blood perfusion as a function of wound healing

**FIGURE 8:**
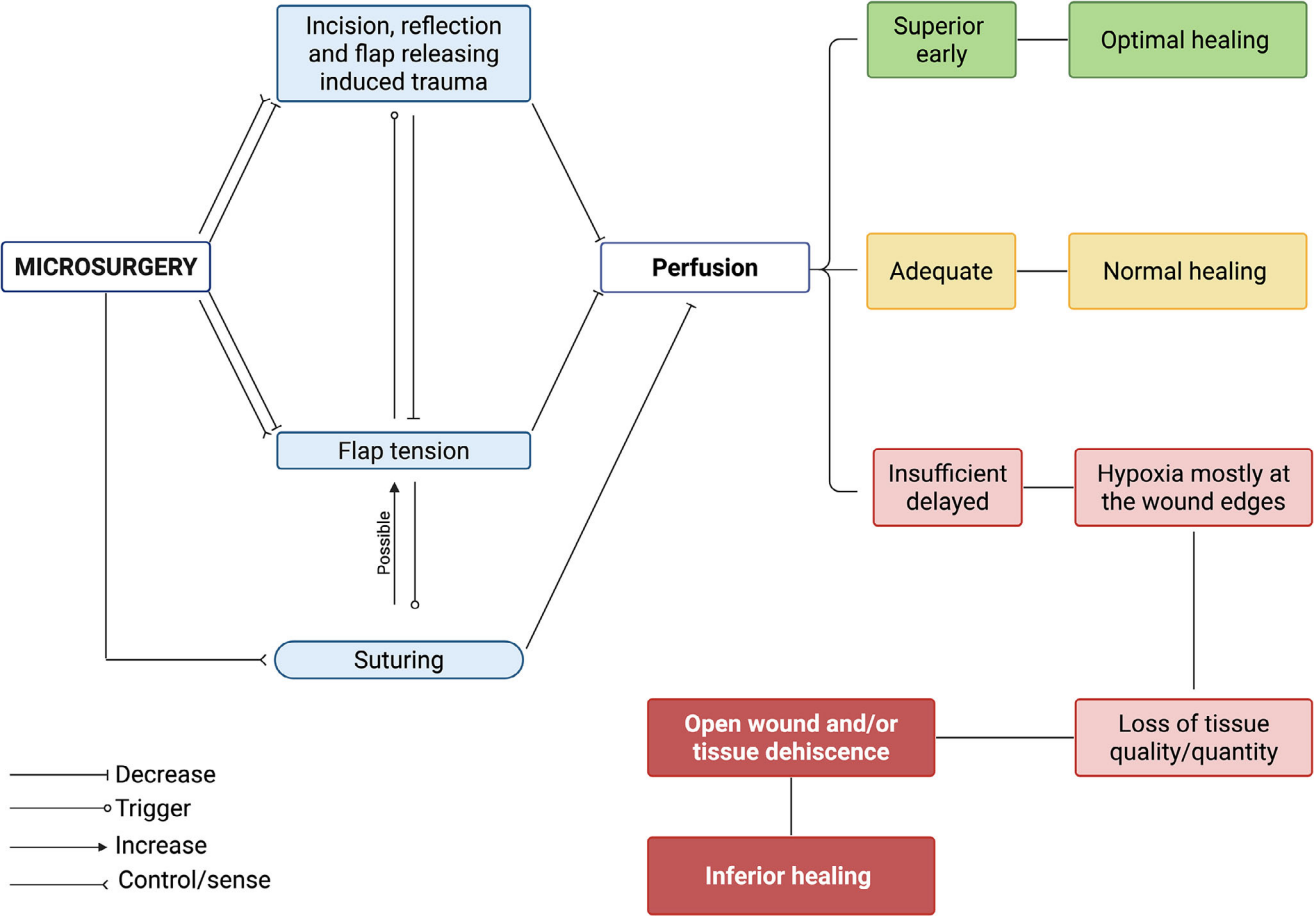
Hypothetical role of microsurgery in tissue perfusion and its impact on wound healing. Microsurgery may modulate blood perfusion through fine balancing surgical trauma, flap tension, and suturing tension, resulting in a higher chance of achieving optimal healing.

Blood perfusion is a key determinant for the success of periodontal and implant‐related procedures, especially hard and soft tissue regenerative procedures.^4^, ^55^ Perfusion refers to the process in which blood carries oxygen and nutrients to tissues and organs and transports waste products of cellular metabolism away.^56^ It is critical because it occurs at the initial phase of wound healing, leading to subsequent proliferative as well as remodeling phases.^11^ Early wound stability primarily relies on sufficient blood perfusion.^57^ Two major factors that impact blood perfusion of a healthy individual are (1) the extent and severity of surgical trauma and (2) the amount of tension within the flap^4^, ^13^ (Figure 8). Conditions that affect systemic perfusion, such as smoking and diabetes are not in the scope of this discussion. Trauma to the microvasculature because of surgical incisions, flap reflection, and flap releasing steps can negatively affect blood perfusion, especially in uncontrolled and indiscriminate manners.^28^ The ratio of the length of vertical incisions in relation to the base of the flap width relates to the amount of revascularization.^9^, ^39^ Flaps with longer vertical incisions showed poorer revascularization. Simply elevating a mucoperiosteal flap can reduce 50% of the baseline perfusion at 24 h. Residual flap tension is correlated with soft tissue hypoxia and ischemia.^58^, ^59^ The stress is exaggerated at the weakest link of the wound system, which is most commonly at the wound edge and thin tissues.^59^ Regenerative procedures often involve placement of autogenous tissues or biomaterials underneath the flap to augment tissue volume.^60^ To release the tension for primary wound closure, flap releasing methods are implemented.^61^ Therefore, flap tension and surgical trauma are inter‐related in the context of blood perfusion.^59^ Presence of residual tension triggers the addition of (an) incision(s) and/or more aggressive release measures. These procedures may reduce the tension but at the same time compromise blood perfusion because of generation of surgical trauma.^59^ Care should be exercised to achieve a balance between flap manipulation and tension minimization for the maintenance of adequate blood perfusion^56^ (Figure 8).

Depending on the amount and the speed of perfusion determined by tissue trauma and tension, wound healing could be arbitrarily classified as (1) optimal, (2) normal, or (3) inferior. In reality a given wound falls into a wide spectrum of healing outcomes.^11^, ^12^, ^55^, ^58^, ^59^, ^62^ Optimal healing leads to a fast transition from the inflammatory stage to the next phases, which may infer enhanced clinical as well as patient‐centered outcomes. On the other hand, inadequate perfusion often time relates to delayed wound healing and inferior outcomes.^55^ Since the wound edge suffers the most from ischemia, tissue loss is a consequence, leading to wound opening and partial or total loss of the grafted materials.^4^, ^9^, ^55^, ^63^ In addition to postoperative perfusion, baseline perfusion and vasculature distribution are equally important since studies have shown a random distribution of the supraperiosteal plexus in oral mucosa and variability in the population.^9^, ^11^, ^27^, ^39^, ^62^, ^64^ In other words, periodontal microvasculature is site as well as individual specific.^63^ This can explain in part why regenerative/reconstructive outcomes may not be as predictable even though known systemic and local contributing factors are controlled in combination with skillful execution.^39^ Many aspects of oral wound healing remain poorly understood. Therefore, assessing the speed and volume of blood perfusion, both at baseline and postoperatively, using non‐invasive imaging techniques could potentially provide a foundation for a comprehensive evaluation and prediction of wound healing outcomes following periodontal and peri‐implant regenerative procedures.

### Hypothesis on optimal microsurgical wound healing

Through microsurgery, early re‐establishing of blood perfusion and eventually enhancing the outcome of root coverage procedures is evident.^3^, ^8^ Therefore, the central hypothesis is that microsurgery encourages early blood perfusion by inducing less surgical trauma and managing flap tension more favorably^1^, ^2^, ^3^, ^58^, ^65^ (Figure 8). However, this hypothesis remains theoretical, and evidence‐based conclusions are necessary to support its validation in future studies. Surgical trauma could be minimized by the minimally invasive approach made possible with high magnification and co‐axial illumination.^5^, ^66^, ^67^ This implies that fewer incisions, less flap extension and reflection are required for the same access needs.^9^ The flap releasing step, if indicated, can be handled in a highly controlled manner under magnification. Controlled and precise microscopic partial thickness incisions on periosteum and connective tissue dissection within the soft tissue flap for tension releasing reduce unnecessary trauma.^3^, ^5^, ^66^ If possible, the surgeon should inform themselves about vascular activity before incision, to potentially avoid severing the most dominant vessels. Fine psychomotor coordination made possible by bimanual instrumentation can deliver an ultimate balance between tissue tension reduction and flap releasing trauma. This bimanual instrumentation refers to the use of micro‐tissue forceps on the non‐dominant hand to grasp the flap and a scalpel on the dominant hand to dissect the flap.^5^, ^66^ The magnitude of force applied to the flap from both hands through the instruments can be sensed and adjusted to be minimal.^59^ Any early signs of tissue dehiscence, for example, the instrument shows through the flap, can be observed under the microscope, with that information, the amount and direction of the exerted force can be modified. Furthermore, flap tension is gauged through micro sutures. Minimal residual tension (up to 0.1 N or 10 g) is desirable for sustained primary wound closure.^59^ The tensile strength of 7‐0 polypropylene sutures is measured at approximately 3.7 N (370 g), respectively.^59^ Therefore, when flap tension exceeds 370 g during the use of 7‐0 sutures, the suture will break. This mechanism serves as a safeguard for avoiding overtightening and unwanted flap tension. Under high magnification, the final flap position can be finely adjusted by controlling the suture tension. These microsurgical advantages collectively might have contributed to the improved clinical outcome observed in the literature through preserving microvasculature by effectively controlling surgical trauma and managing flap tension, leading to optimal blood perfusion postoperatively. The healing outcomes seen in daily clinical care are arbitrarily defined as optimal, normal, or inferior for discussion purposes (Figure 6). In reality, the healing outcome can fall into a wide spectrum in between the two extremes. Microsurgery may shift the proportion of its outcome toward optimal or deliver more consistent and predictable outcomes, resulting in significant improvement at the statistical scale. Therefore, the identified high‐frequency dental US and LSCI stand out as promising methods for studying the possible intercorrelations between surgical trauma, flap tension, blood perfusion, and clinically measurable outcomes.

## CONCLUSION

While fundamentally different, with LSCI assessing superficial tissue perfusion over a broader region and US capturing deeper perfusion in cross‐sectional images, both techniques can complement each other to provide valuable insight into the spatial and temporal dynamics of blood perfusion, an essential part of healing. In addition, high‐frequency dental US and LSCI stand out as promising methods for studying the possible intercorrelations between surgical trauma, flap tension, blood perfusion, and clinically measurable outcomes. Importantly, current diagnostic frameworks for periodontal and peri‐implant diseases continue to rely on late‐stage markers such as probing depth PD, BOP, and bone loss. To fully realize the potential of LSCI and US as non‐invasive tools for early diagnosis and microsurgical guidance, further validation through large‐scale, controlled human studies is essential, alongside the development of updated diagnostic protocols.

## AUTHOR CONTRIBUTIONS

*Conceptualization, methodology, software, formal analysis, data curation, writing—original draft, and writing—review and editing*: Amanda B. Rodriguez. *Conceptualization, methodology, software, formal analysis, data curation, writing—review and editing, project administration, and funding acquisition*: Oliver D. Kripfgans. *Conceptualization, methodology, software, formal analysis, data curation, writing—original draft, and writing—review and editing*: Diego Velásquez‐Plata. *Conceptualization, methodology, software, formal analysis, data curation, writing—original draft, writing—review and editing, project administration, and funding acquisition*: Hsun‐Liang Chan.

## CONFLICT OF INTEREST STATEMENT

The authors declare they have no conflicts of interest.

## PATIENT CONSENT FOR PUBLICATION

The clinical case described in this report was collected as part of a feasibility study approved by the University of Michigan Medical School Institutional Review Board (HUM00203875). All study procedures complied with the Declaration of Helsinki, and written informed consent was obtained from the participant. No patient‐identifiable information is contained in this case report/series.
